# No Causal Association Between Coffee Consumption and Risk of Migraine: A Mendelian Randomization Study

**DOI:** 10.3389/fgene.2022.792313

**Published:** 2022-01-25

**Authors:** Heng Chen, Han Zhang, Liangrong Zheng

**Affiliations:** Department of Cardiology, The First Affiliated Hospital, College of Medicine, Zhejiang University, Hangzhou, China

**Keywords:** Mendelian Randomization, causal association, coffee consumption, any migraine, migraine with aura, migraine without aura

## Abstract

**Background and Aims:** Coffee consumption has been suggested to increase the risk of migraine. However, causality remains inconclusive. In the present study, we performed a two-sample Mendelian randomization (MR) analysis to investigate the causal relationship between coffee consumption and migraine.

**Methods:** We obtained nine single-nucleotide polymorphisms (SNPs) associated with coffee consumption at genome-wide significance (*p* < 5 × 10^−8^) from a large genome-wide association study (GWAS) based on the UK Biobank study (included 375,833 individuals). Summary-level data for any migraine (AM) and its subtypes (migraine with aura (MA) and migraine without aura (MO)) were obtained from the largest available GWAS of migraine conducted by the International Headache Genetics Consortium (IHGC) (included 59,674 cases and 316,078 controls). MR estimates were pooled using fixed-effect inverse-variance weighted (IVW) as the main method. Sensitivity analyses were further performed using weighted median, MR-Egger, and MR-PRESSO to assess the robustness of our findings.

**Results:** Genetically-predicted 50% increase of coffee consumption was not causally associated with the risk of AM (odds ratio (OR), 0.97; 95% confidence interval (CI), 0.83–1.14; *p* = 0.71), MA (OR, 0.81; 95%CI, 0.58, 1.12; *p* = 0.19), or MO (OR, 0.97; 95%CI, 0.72, 1.30; *p* = 0.83) in the fixed-effect IVW methods. Sensitivity analyses returned similar results. No directional pleiotropy was found.

**Conclusion:** This MR study does not support a causal relationship between genetically predicted coffee consumption and the risk of migraine. Coffee consumption is likely not a trigger nor a prevention strategy for migraine headaches.

## Introduction

Migraine is a disease with unilateral, pulsating, activity-aggravated headache lasting for 4–72 h accompanied by nausea, phonophobia, photophobia, or both (Goadsby et al., 2017). In 2016, ∼1.04 billion people worldwide suffered from migraines (Burch et al., 2019). Moreover, migraine remains the sixth largest cause of years lived with disability in the world (Collaborators, 2017).

Coffee consumption has been associated with migraine for many years (Bigal et al., 2002; Takeshima et al., 2004; Hagen et al., 2009; Tai et al., 2018) and was suggested as one of the dietary triggers for the disorder (Mollaoğlu, 2013; Zaeem et al., 2016; Tai et al., 2018). On the other hand, accumulating evidence demonstrated that caffeine, as an analgesic adjuvant, can reduce pain sensation during migraine attacks (Derry et al., 2014; Baratloo et al., 2017). Since available data on coffee consumption and migraine risk come from observational studies that may be influenced by biases such as residual confounding, misclassification, and reverse causation (Smith and Ebrahim, 2003), causality in the association remains inconclusive. A question arises: is coffee consumption a trigger for migraine, or is the association entirely due to the fact that migraineurs are more likely to drink coffee to relieve the pain? Given that coffee consumption is one of the most common modifiable exposures, recognition of the causal link may advance the development of the preventive strategy for migraine.

Mendelian randomization (MR) study is an approach to investigate the causal relationship between exposures and outcomes by using germline genetic variants as instrumental variables (IVs) (Lawlor et al., 2008). The method is considered as a “nature” RCT and diminishes confounding and reverse causation in observation research (Lawlor et al., 2008). Therefore, the single-nucleotide polymorphisms (SNPs) influencing coffee consumption can be used to analyze the association. In the present study, we aimed to use the two-sample MR analysis to determine the causal effect of coffee consumption on any migraines (AM), migraines with aura (MA), and migraines without aura (MO).

## Methods

### Study Design

**FIGURE 1:**
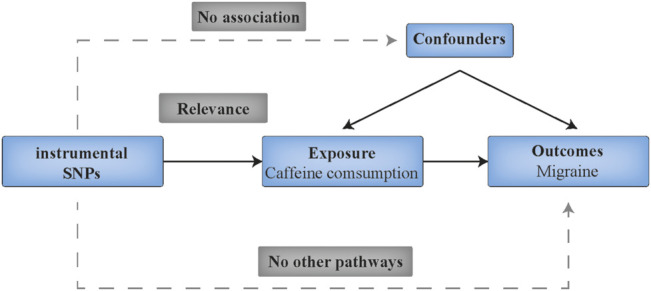
Schematic diagram showing the assumptions of Mendelian randomization analysis. SNPs indicates single nucleotide polymorphisms.

This study followed the Strengthening the Reporting of Observational Studies in Epidemiology Using Mendelian Randomization (STROBE-MR) guide (Skrivankova et al., 2021). A diagram of this two-sample MR analysis is displayed in Figure 1. Specifically, the analysis is based on the following key assumptions: (1) the IVs (SNPs) should be robustly associated (*p* < 5 × 10^−8^) with the exposure (coffee consumption); (2) the IVs should be independent of any potential confounders; and (3) the IVs should not directly affect the outcome (migraine) except through their effect on the exposure.

### Data Sources and SNP Selection

The current MR analyses were performed using published GWAS datasets (Supplementary Table S1). No specific ethical approval and written informed consent of participants is required.

Genetic IVs for coffee consumption were obtained from the largest available genome-wide association study (GWAS) meta-analysis using the UK Biobank Resource (Zhong et al., 2019). The UK Biobank study included over 500,000 participants aged 37–73 years from 22 centers across England, Wales, and Scotland in 2006–2010. In this study, 375,833 samples were included after the exclusion of outliers based on heterozygosity and missingness, individuals with sex discrepancy between self-reported and inferred sex, and closely related individuals (kinship coefficient of >0.0442). In addition, the analysis was restricted to those who self-reported as “British” and shared similar ancestral backgrounds (Zhong et al., 2019). Self-reported coffee consumption was retrieved from all participants based on the question “How many cups of coffee do you drink each day (include decaffeinated coffee)?” (Zhong et al., 2019). The authors identified 15 SNPs with genome-wide significance (*p* < 5 × 10^−8^) (Zhong et al., 2019). To select valid instrumental SNPs, we first pruned SNPs with horizontal pleiotropic effects to meet the second key assumption–the IVs should be independent of any potential confounders. By searching the PhenoScanner database (Kamat et al., 2019), 5 SNPs were removed for being associated with potential confounders (*p* < 5 × 10^−8^), such as body mass index and alcohol consumption (Supplementary Table S2). Second, we excluded rs4719497 since it was in linkage disequilibrium (r^2^ < 0.01; region size, 10000 kb) with other SNPs (rs4410790 and rs12699844). Finally, nine coffee-associated SNPs were used as IVs for the MR analyses (Supplementary Table S3). F-statistics were calculated to assess the strength of each SNP using the formula $F=R^{2}\frac{N-2}{1-R^{2}}$ . Here R^2^ indicates the proportion of the variance of coffee consumption explained by the IVs, and N refers to the total sample size (Burgess and Thompson, 2011). No proxy-SNPs were necessary since all the coffee-related SNPs were available in the outcome datasets.

Summary statistics for the associations of the coffee-related SNPs with migraine were derived from the hitherto largest GWAS meta-analysis of migraine conducted by the International Headache Genetics Consortium (IHGC), which comprised data from 22 studies with a total of 59,674 cases and 316,078 controls of European ancestry (Gormley et al., 2016). Migraine was defined based on previously published diagnostic criteria from the International Headache Society. (2013). Two prevalent sub-forms of migraine were also included in the present study: MA (included 6,332 cases and 144,883 controls) and MO (included 8,348 cases and 139,622 controls) (Supplementary Table S1).

### Statistical Analysis

After harmonizing the SNPs across the data sources via the effect alleles, we calculated the effect estimate for each instrumental SNP on migraine with the Wald estimator, and assessed the possible measurement errors using the Delta method (Lawlor et al., 2008). The fixed-effects inverse variance-weighted (IVW) method was used as standard analysis to derive the final effect estimates. Heterogeneity among estimates of SNPs was measured by Cochran Q-derived p, I^2^, and the funnel plot (Sterne et al., 2011; Greco M et al., 2015). Sensitivity analyses included the multiplicative random-effects IVW(Bowden et al., 2017), the weighted median (Bowden et al., 2016), the MR-Egger regression method (Burgess and Thompson, 2017), and the MR-pleiotropy residual sum and outlier (MR-PRESSO) method (Verbanck et al., 2018). Where heterogeneity existed (I^2^ > 25% or Cochran Q-derived *p* < 0.05) (Greco M et al., 2015), the multiplicative random-effects IVW method was adopted to avoid the bias of weak SNP-exposure associations (Bowden et al., 2017). The weighted median method can provide valid estimates even when up to 50% of the information in the analysis comes from invalid IVs(Bowden et al., 2016). The MR-Egger method provides more conservative causal estimates in the presence of pleiotropic variants and is less likely to generate inflated test statistics (Burgess and Thompson, 2017). The MR-PRESSO method was used to detect the presence of outliers that could bias the results (Verbanck et al., 2018). We applied the intercept test from MR-Egger to assess horizontal pleiotropy (Bowden et al., 2015). In addition, we employed leave-one-out analyses to determine whether a single SNP drove the causal relationship. With this approach, we excluded one SNP in turn and then re-evaluated the causal effect. Scatter plots depicting the associations were also provided.

The OR estimates of AM, MA, and MO were scaled per 50% increase in coffee consumption (0.5 more cups of coffee). Two-sided p values of < 0.0167 (= 0.05/3 outcomes) were set as the thresholds for significance. Statistical power was calculated with an online tool (https://shiny.cnsgenomics.com/mRnd/) (Brion et al., 2013). All MR analyses were conducted using R software (version 4.1.0) with R packages including TwoSampleMR (Hemani et al., 2018), MendelianRandomization (Yavorska and Burgess, 2017), and MR-PRESSO(Verbanck et al., 2018).

## Results

All IVs were estimated to account for 0.5% of the observed variance of coffee consumption. None of these IVs had an F-statistic lower than the threshold of 10, suggesting no weak instrument bias in the present study (Supplementary Table S3). Our MR analyses had over 80% statistical power to detect an odds ratio (OR) of 1.17 (or 0.83) for the coffee consumption-AM relationship, 1.49 (or 0.51) for the coffee consumption-MA relationship, and 1.43 (or 0.57) for the coffee consumption-MO relationship.

**FIGURE 2:**
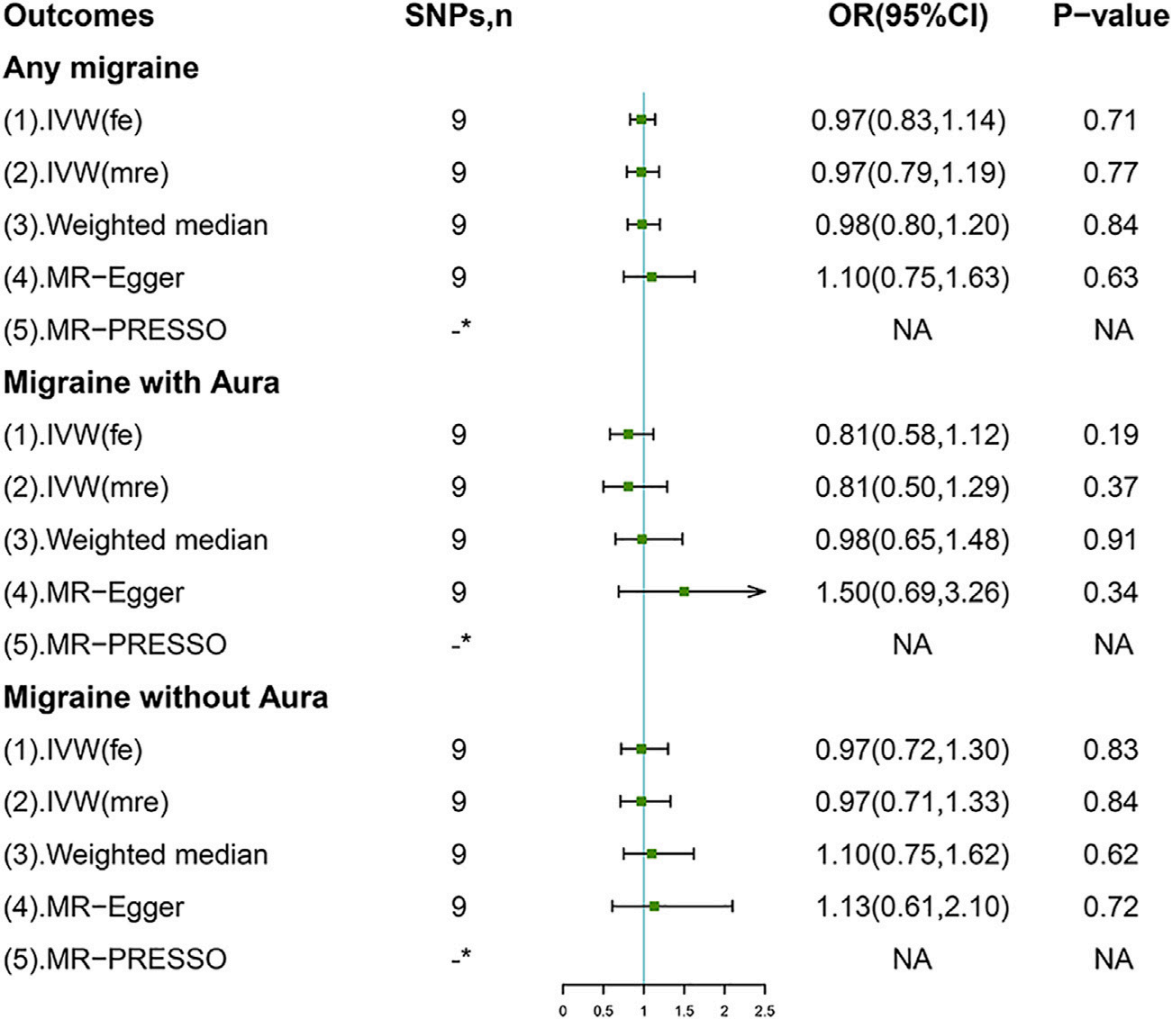
Associations of genetically predicted coffee consumption with migraine and its subtypes. IVW (fe), fixed-effects inverse-variance weighted; IVW (mre), multiplicative random-effects inverse-variance weighted; MR-Egger, Mendelian randomization-Egger; MR-PRESSO, Mendelian randomization pleiotropy residual sum and outlier method; NA, not available. *No outliers detected.

In the standard IVW method, genetically-predicted 50% increase of coffee consumption was not associated with the risk of AM (OR, 0.97; 95% confidence interval (CI), 0.83–1.14; *p* = 0.71), MA (OR, 0.81; 95%CI, 0.58, 1.12; *p* = 0.19), or MO (OR, 0.97; 95%CI, 0.72, 1.30; *p* = 0.83) (Figure 2). No outliers were detected with the MR-PRESSO test (Figure 2). However, there was some evidence of heterogeneity in the IVW analyses as demonstrated by Cochran Q-derived p, I^2^ (Supplementary Table S4), and funnel plots (Supplementary Figure S1); thereby we applied the multiplicative random-effects IVW method, which yielded similar results (Figure 2). The scatter plots for AM, MA, and MO are displayed in Supplementary Figure S2. Forest plots of the effect of each single SNP on the outcomes are provided in Supplementary Figure S3. However, we may not have reached sufficient statistical power to detect such weak associations.

Sensitivity analyses including weighted median and MR-Egger methods returned consistent results (Figure 2). Leave-one-out analyses indicated the non-causal associations were not biased by any single genetic variant (Supplementary Figure S4). Importantly, no evidence of directional pleiotropy was found as measured by the MR-Egger intercept and the MR-PRESSO global test (p for intercept > 0.264; p for global test > 0.075; Supplementary Table S4).

## Discussion

The present study reveals no causal effect of the genetic increase in coffee consumption on any type of migraine, and the results are consistent across sensitivity analyses. To our knowledge, this is the first MR study to explore the causal association between coffee consumption and the risk of migraine.

Previous observational studies have reported the association between regular coffee consumption and migraine. A prospective cross-sectional study demonstrated that coffee consumption is significantly associated with migraine prevalence (OR, 1.73; 95% CI, 1.12–2.68; *p* = 0.014) (Tai et al., 2018). In the Head-Hunt study, after adjusting for confounders such as age, gender, smoking, and level of education, coffee consumption is accompanied by an increased migraine incidence (OR, 1.13; 95% CI, 1.07-1.20; *p* < 0.05) (Hagen et al., 2009). Similarly, findings from a randomized case-control study revealed a positive correlation between coffee consumption and chronic migraine (OR, 2.9; 95% CI, 1.5–5.3; *p* < 0.0001) (Bigal et al., 2002). These findings suggested that coffee may act as a trigger for migraine. The prevalence of coffee as a migraine trigger ranges from 6.3 to 14.5% (Zaeem et al., 2016). On the other hand, coffee was suggested as an analgesic adjuvant for migraine. A double-blind RCT study showed that pain relief after caffeine monotherapy is faster than after placebo treatment (Diener et al., 2005). Mechanically, caffeine can inhibit NO synthase production and produce cerebral vasoconstriction, thereby reducing pain sensation during migraine attacks (Nowaczewska et al., 2020). Therefore, it is also possible that migraineurs drink coffee to relieve the pain, leading to the wrong conclusion in traditional studies.

In this study, we aimed to determine whether coffee consumption, as one of the most common modifiable exposures, is associated with higher migraine risk. However, the analyses did not suggest a causal association of coffee consumption with migraine risk as anticipated. This can be explained from the following perspectives. First, caffeine has been suggested as an analgesic adjuvant for migraine; migraineurs tend to drink coffee to relieve the pain, leading to a cause-effect inversion bias in traditional studies. Second, although some confounders in observational studies were adjusted, unmeasured risk factors cannot be completely ruled out. Third, it is well-known that migraines are usually preceded by some premonitory symptoms. Drinking coffee might simply be a consequence of premonitory symptoms that herald a headache. Food craving and other symptoms like yawning and sleepiness in the premonitory phase may be responsible for drinking coffee, leading to a false connection between coffee consumption and migraine (Nowaczewska et al., 2020). Our results were in agreement with a prospective cross-sectional study in which no subjects reported coffee as a trigger for migraine (Yadav et al., 2010).

The Mendelian randomization design is one of the major strengths of this study. By using randomly allocated genetic variants as IVs, our study largely mitigated confounding or reverse causation bias, thus providing compelling evidence. In addition, since the analyses were restricted to individuals of European ancestries, the bias introduced by population structure was unlikely to affect our results. Other strengths included the stability of the causal estimates across different sensitivity analyses, the large sample size derived from several GWAS datasets, and the strong estimated effects of each IVs (all F-statistic >10).

There are several limitations in our study. First, we are unable to explore the potential non-linear association between coffee consumption and migraine since this study was based on summary-level data. Second, since the population in this study was restricted to Europe, we are not sure if the same conclusion can be reached in non-European populations. Third, the statistical power for the present study may be insufficient since only 0.5% of the observed variance in coffee consumption was explained by IVs. Therefore, we should be cautious with interpreting the negative results; the null association might be due to a lack of power. Fourth, data on self-reported coffee consumption might be imprecise and is likely to introduce measurement bias. In addition, coffee consumption may not be very highly heritable; it is commonly not a life-long exposure, which reduced the clinical relevance of this MR study. Finally, potential directional pleiotropy that may bias estimation of causal inference cannot be completely ruled out, despite the lack of evidence from MR-Egger regression and MR-PRESSO methods.

## Conclusion

This MR study provides no evidence of a causal association between increased coffee consumption and the risk of migraine in European populations. Coffee consumption is likely not a trigger nor a prevention strategy for migraine headaches.

## Data Availability

The original contributions presented in the study are included in the article/Supplementary Material, further inquiries can be directed to the corresponding author.
